# The role of NT-proBNP in screening for atrial fibrillation in hypertensive disease

**DOI:** 10.1016/j.ijcha.2024.101549

**Published:** 2024-11-08

**Authors:** Gina Sado, Katrin Kemp Gudmundsdottir, Carl Bonander, Mattias Ekström, Johan Engdahl, Emma Svennberg

**Affiliations:** a Karolinska Institutet, Department of Medicine, Karolinska University Hospital Huddinge, Sweden; b Karolinska Institutet, Department of Clinical Sciences – Danderyd University Hospital, Sweden; c School of Public Health and Community Medicine, Institute of Medicine, University of Gothenburg, Sweden; d Department of Cardiology, Danderyd University Hospital, Stockholm, Sweden

**Keywords:** Atrial fibrillation, Hypertension, Hypertension grades, Blood pressure, NT-proBNP, Mass screening

## Abstract

**Background:**

Atrial fibrillation (AF) screening should be considered in elderly patients with high risk of stroke, which include individuals with hypertension. The biomarker N-terminal prohormone of brain natriuretic peptide (NT-proBNP) can predict incident AF and is increased in hypertensive individuals. The aim of this study is to investigate the incidence of screening-detected AF in elderly individuals in relation to NT-proBNP and hypertension.

**Methods:**

STROKESTOP II is a randomized controlled trial in which 75/76-years-old individuals were invited to a screening study for AF using NT-proBNP as a discriminator of high risk. In this sub-study, a prior hypertension diagnosis was self-reported by participants and measured blood pressure was stratified into hypertension-grades. Individuals with both increased blood pressure (≥140 mmHg) and NT-proBNP ≥ 125 ng/L were defined as a high-risk group. The lowest risk-group was defined as normotensive participants with NT-proBNP < 125 ng/L.

**Results:**

NT-proBNP increased gradually for every hypertension-grade above hypertension-grade 1 compared to normotensive participants. Screening-detected AF was most common in normotensive participants with increased NT-proBNP (n = 90/1922, 4.7 %), followed by patients with both NT-proBNP > 125 ng/l and SBP ≥ 140 mmHg, (AF = 65/1741, 3.7 %) compared to the low-risk group (AF = 2/1444, 0.1 %), p < 0.001.

**Conclusion:**

NT-proBNP is elevated in elderly patients with hypertension and increases with grades of hypertensive disease. NT-proBNP is a strong predictor of AF regardless of high blood pressure, and the risk for screening-detected AF is very low in participants with normal blood pressure and low NT-proBNP. A combination of blood pressure and NT-proBNP could identify suitable participants for AF screening.

## Highlights

• The risk of screening-detected AF is low in individuals with normal blood pressure and low NT-proBNP.
• Screening-detected AF is highest in individuals with only elevated NT-proBNP.
• NT-proBNP is elevated in elderly patients with hypertension and increases with grades of hypertensive disease.
• NT-proBNP could in the future work as a predictive tool in hypertensive individuals for AF screening.

## 1 Introduction

Atrial Fibrillation (AF) is a common arrhythmia that is associated with an increased risk of ischemic stroke [1]. AF can be difficult to diagnose due to its asymptomatic or intermittent character [2]. Cardiac biomarkers, such as N-terminal prohormone of brain natriuretic peptide (NT-proBNP) and troponin, have been studied in conjunction with AF. Several studies have showed that NT-proBNP can work as a possible additive risk marker for stroke and a possible predictor for AF [3], [4], [5], [6], [7].

NT-proBNP is elevated in patients with AF and is a promising marker in detecting AF [4], [6], [8], [9], [10], [11]. The biomarker is easily analyzed by blood tests, easily obtained and used in clinical practice [12]. In past studies [13], [14], NT-proBNP remained elevated in patients with AF after adjusting for presence of heart failure and other cardiac diseases, including hypertension and coronary heart disease. In addition, NT-proBNP levels decrease after successful cardioversion from AF to sinus rhythm [15], [16], [17]. Hence, NT-proBNP could be a future diagnostic/prognostic tool, possibly used together with other analyses/scores, in predicting those at high risk for AF. In the STROKESTOP II trial, a large population-based screening study for AF, NT-proBNP was used as a possible discriminator to identify a population at high risk of future AF [9].

AF and hypertension often coexist [18]. Hypertensive disease is a main contributor to AF [19] and among individuals who develop AF, hypertension is present in 60–80 % [20]. However, there is still a lack of knowledge if the risk of incident AF increases with grades of hypertension, and if adequate blood pressure control decreases the risk of AF in individuals with sinus rhythm [21], [22], [23]. In addition, little is known about the association between NT-proBNP and blood pressure, and if NT-proBNP could serve as a diagnostic/prognostic tool in differentiating whom to screen for incident AF, in a population with hypertension.

The aim of this sub study of the STROKESTOP II trial, was to investigate the levels of NT-proBNP in elderly patients with hypertension/hypertension-grades and to determine if the incidence of screening-detected AF increases with high blood pressure and NT-proBNP levels (high-risk group), compared to normal blood pressure/NT-proBNP levels.

## 2 Methods

### 2.1 Study population and diagnostic methods and definitions

This is a sub-study of the population screening study STROKESTOP II [9], a Swedish study in which NT-proBNP was used together with ECG-screening for detecting AF. All 75/76-year-old individuals (n = 28 712) living in the Stockholm region were identified by their personal identification number through Statistics Sweden, and randomized 1:1 to be invited to a screening program for AF or to serve as a control group [9]. In this sub-study, the findings of the participants were reported immediately after the screening program as a cross-section in time.

All participants were invited to an index visit for analyzing NT-proBNP in venous blood, single-lead ECG-screening, measuring blood pressure and for reporting their medical history. Participants’ self-reported medical history included prior AF-, stroke/transient ischemic attack (TIA), myocardial infarction diagnosis, presence of peripheral arterial artery disease, hypertensive disease, diabetes, heart failure or a pacemaker, treatment with oral anticoagulants, as well as palpitation symptoms and weight/height.

In participants without known AF, NT-proBNP was analyzed from venous blood samples using point-of-care analysis (Cobas 232, Roche diagnostics, Rotkreutz, Switzerland). Participants were stratified into two groups, a high-risk group with NT-proBNP ≥ 125 ng/L and a low risk-group with NT-proBNP < 125 ng/L. The cut-off levels were based on previous findings [24]. An index ECG-recording consisting of a 30-second handheld single-lead device (Zenicor One, Zenicor Medical Systems, Stockholm, Sweden) was used in all participants without prior AF, regardless of NT-proBNP levels. For the high-risk group, an extended 2-week intermittent handheld ECG-recording 4 times a day was performed unless AF was diagnosed at index ECG. No further examination was done in the low-risk group if the index ECG-recording showed sinus rhythm. In patients with known AF on oral anticoagulant treatment, no further examination was done.

Blood pressure was measured after a few minutes with the participant in a sitting position by a trained nurse through an automatically blood pressure cuff (Omron M6 Comfort IT). At the baseline visit, if blood pressure was higher than 140/90 mmHg, a repeated measurement was taken. If blood pressure was normal (<140/90 mmHg), the blood pressure measurement was not repeated. The last measurement was registered. In case blood pressure remained elevated, participants with high blood pressure with and without a prior hypertension diagnosis were referred to their general practitioner or a cardiologist for follow up. Elevated systolic blood pressure (SBP) was graded according to ESC Guidelines, normal blood pressure (<140 mmHg), hypertension grade 1 (140–159 mmHg), hypertension grade 2 (160–179 mmHg) and hypertension grade 3 (≥180 mmHg), regardless of hypertension diagnosis [25], [26].

Incident AF was defined as at least one episode of irregular rhythm with a duration of at least 30 s on the single-lead ECG. Patients with SBP ≥ 140 mmHg and NT-proBNP  ≥ 125 ng/L was defined as a high-risk group. The lowest risk-group was defined as participants with a normal SBP combined with NT-proBNP levels < 125 ng/L.

Patients with a known diagnosis of heart failure and AF according to their self-reported diseases statement were excluded from this analysis.

### 2.2 Statistics

Categorical variables in baseline characteristics are presented as counts and percentages, and chi-squared tests were used to compare differences in groups. Continuous variables are presented as median and IQR.

NT-proBNP had a skewed distribution. The comparison between the hypertensive and non-hypertension groups were therefore analyzed using a Mann-Whitney *U* test. For comparison of NT-proBNP across the four hypertension grades, a Kruskal-Wallis test was employed. Linear regression of log-transformed NT-proBNP, which was normally distributed, was used to adjust for sex, height, weight, and comorbidities such as diabetes, vascular disease, previous stroke, incident AF, and hypertension. The evaluation of incident AF in higher SBP and elevated NT-proBNP, compared to lower blood pressure and NT-proBNP, was assessed through chi-squared tests comparing the high-risk and low-risk groups.

Two-sided p-values < 0.05 were considered statistically significant. All statistical tests were performed with IBM SPSS Statistics V.29 software.

### 2.3 Ethics

The study complies with the Declaration of Helsinki, and the protocol was approved by the regional ethics committee (2015/2079–31/1 and 2017/974–32). All participants provided informed consent.

## 3 Results

### 3.1 Study population

There were 14 304 remaining participants suitable for screening. The mean age of the participants was 75 years and 55.5 % were women. After exclusion of non-participants (n = 6 529), individuals who died or emigrated (n = 907), and participants with known AF (n = 553), 6 315 participants remained in the study. After exclusion of individuals with missing values of NT-proBNP (n = 24) and participants with a known diagnosis of heart failure (n = 78), 6 213 participants with reported blood pressure status and a valid NT-proBNP value remained for analysis (Supplementary Figure A.1).

### 3.2 NT-proBNP in hypertension and AF

In total, 3 169 (51 %) of the study participants had a self-reported diagnosis of hypertension. The mean SBP was 135 mmHg (IQR 124–144) in participants without a known diagnosis of hypertension, and 142 mmHg (IQR 131–156) in participants with hypertension, p < 0.001. In total, there were 157 participants (2.5 %) with screening-detected AF. There were no significant differences in screening-detected AF between the group with (n = 82, 2.6 %) and without (n = 72, 2.3 %) self-reported hypertension, p = 0.573.

In the entire cohort (n = 6 213), median NT-proBNP was 156 ng/L in the hypertension-group compared to 144 ng/L in the non-hypertension group, p = 0.014 (Table 1). This remained significant even if patients with screening-detected AF were excluded (median NT-proBNP 154 ng/L vs 142 ng/L respectively, p = 0.025). In the group with screening-detected AF (n = 157), median NT-proBNP was significantly higher (median NT-proBNP 318 ng/L vs 148 ng/L) than the group without screening-detected AF, p < 0.001 (Table 2). No difference in NT-proBNP levels was observed in patients with incident AF regardless of presence or absence of hypertension (Fig. 1A)*.*

**Table 1:** Baseline characteristics in 75/76-year-old participants stratified by self-reported hypertension/non-hypertension.

	**Non-hypertension n = 3044**	**Hypertension n = 3169**	**p-value**
**Sex, n (%)**			
Female	1753 (57.6 %)	1697 (53.6 %)	0.001
			
**Comorbidities, n (%)**			
Diabetes	173 (5.6 %)	494 (15.6 %)	<0.001
Prior stroke/TIA	152 (5 %)	290 (9.2 %)	<0.001
Vascular disease	101 (3.3 %)	254 (8.1 %)	<0.001
			
**Median (IQR)**			
NT-proBNP, ng/L	144 (86–249)	156 (83–274)	0.014
CHA_2_DS_2_-VASc score	3 (2–3)	4 (3–4)	<0.001
Systolic BP,mmHg	135 (124–144)	142 (131–156)	<0.001
Diastolic BP,mmHg	81 (74–87)	82 (75–89)	<0.001
Height, cm	170 (163–177)	170 (163–176)	0.153
Weight, kg	71 (63–80)	75 (66–85)	<0.001
Body mass index, kg/m^2^	24.4 (22.2–26.8)	25.9 (23.7–28.7)	<0.001
			

There were 24 missing values regarding self-reported hypertension/non-hypertension diagnosis and were excluded. Three of the excluded participants developed screening-detected AF, n AF = 154. AF, atrial fibrillation; NT-proBNP; N-terminal pro-B-type natriuretic peptide; BP, blood pressure; Transient ischemic attack, TIA. Vascular disease: Myocardial infarction and peripheral vascular disease.

**Table 2:** Baseline characteristics in 75/76-year-old participants stratified by screening-detected AF.

	**No AF n = 6080**	**AF n = 157**	**p-value**
**Sex, n (%)**			
Female gender	3392 (56 %)	1658 (54 %)	0.046
			
**Comorbidities, n (%)**			
Diabetes	655 (11 %)	14 (9 %)	0.469
Prior stroke/TIA	439 (7 %)	7 (4,5%)	0.179
Vascular disease	351 (6 %)	9 (6 %)	0.989
Hypertension	3087 (51 %)	82 (53 %)	0.573
			
**Median (IQR)**			
NT-proBNP, ng/L	148 (83–254)	318 (203–667)	<0.001
CHA2DS2-VASc score	3 (3–4)	3 (3–4)	0.158
Systolic BP, mmHg	138 (128–151)	137 (124–148)	0.099
Diastolic BP, mmHg	81(75–88)	81(74–90)	0.800
Height, cm	169 (163–176)	172 (165–180)	0.003
Weight, kg	73 (64–82.6)	76 (65–85)	0.019
Body mass index, kg/m^2^	25.1(23–27.8)	25.8 (23.1–28.3)	0.391
			

AF, atrial fibrillation; NT-proBNP; N-terminal pro-B-type natriuretic peptide; BP,blood pressure; Transient ischemic attack, TIA. Vascular disease: Myocardial infarction and peripheral vascular disease.

**Fig. 1A:**
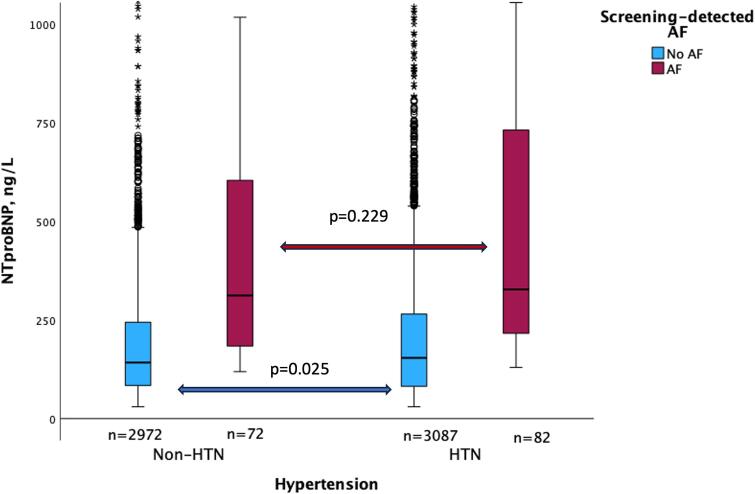
**Boxplots of Median NT-proBNP levels in hypertension/non-hypertension ± AF.** Some outliers were not shown (total NT-proBNP > 1000 ng/L, n = 82 in both groups). AF, atrial fibrillation; HTN, hypertension; non-HTN, non-hypertension; NT-proBNP, N-terminal pro-B-type natriuretic peptide.

Individuals with a hypertension diagnosis had a significantly higher NT-proBNP, overall comorbidities, body mass index (BMI) and weight (Table 1). When adjusted for sex, weight, height, and cardiovascular comorbidities, apart from hypertension, only AF and vascular disease remained positively associated with log NT-proBNP (Supplementary table B2).

### 3.3 NT-proBNP in systolic blood pressure/hypertension grades

Measured SBP from participants were stratified into hypertension grades according to ESC guidelines [25], [26]. When comparing NT-proBNP in the different hypertension-grades, there was an association between hypertension grade and NT-proBNP. NT-proBNP increased for every hypertension-grade except when comparing normal blood pressure and hypertension-grade 1 (Fig. 1B and Table 3). This pattern remained after adjusting for sex, weight, height, blood pressure and cardiovascular comorbidities, regardless of AF (Supplementary Table A.1 and Table B.2).

**Fig. 1B:**
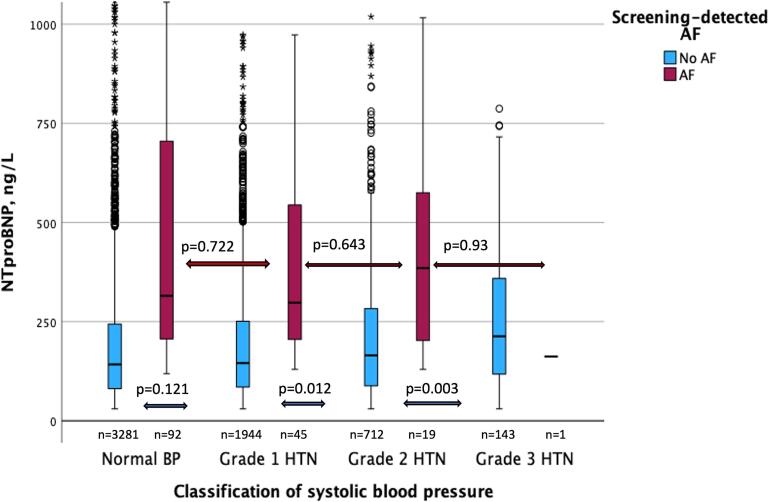
**Boxplots of median NT-proBNP levels in hypertension-grades according to ESC Guidelines ± incident AF.** Some outliers of NT-proBNP were not shown in HTN-grades ± AF (total NT-proBNP > 1000 ng/L, n = 82 in both groups). Normal BP(<140 mmHg), Grade 1 HTN (140–159 mmHg), Grade 2 HTN (160–179 mmHg), Grade 3 HTN (≥180 mmHg). AF, atrial fibrillation; HTN, hypertension; non-HTN, non-hypertension; NT-proBNP; N-terminal pro-B-type natriuretic peptide; BP, blood pressure.

**Table 3:** Characteristics and median NT-proBNP of 75/76-year-old participants with different hypertension-grades.

	**NBP n = 3373**	**HTN grade 1n = 1989**	**HTN grade 2n = 731**	**HTN grade 3n = 144**	**p-value (NBP vs HTN grades)**
**Sex, n (%)**					
Female gender	1981 (58.7 %)	1039 (52.5 %)	356 (48.7 %)	91 (63.2 %)	p < 0.001
**Comorbidities, n(%)**					
Screening-detected AF	92 (2.7 %)	45(2.3 %)	19(2.6 %)	1(0.7 %)	p = 0.376
Diabetes	372 (11 %)	216 (10.9 %)	66 (9 %)	15 (10.4 %)	p = 0.497
Prior stroke/TIA	260 (7.8 %)	138 (7 %)	43 (5.9 %)	5 (3.5 %)	p = 0.093
Vascular disease	204 (6.1 %)	110 (5.5 %)	38 (5.2 %)	8 (5.6 %)	p = 0.759
Hypertension	1392 (41.4 %)	1176 (59.3 %)	493 (67.6 %)	108(75.5 %)	p < 0.001
**Median, (IQR)**					
NT-proBNP, ng/L	145 (82–251)	149 (87–255)	167 (90–292)	213 (118–359)	*
CHA_2_DS_2_-VASc score	3 (3–4)	3 (3–4)	3 (3–4)	4(3–4)	*
Systolic BP,mmHg	128 (120–134)	148 (143–153)	166 (162–171)	185 (182–191)	*
Diastolic BP, mmHg	77 (71–82)	85 (79–91)	91 (84–97)	97 (90–103)	*
Height, cm	169 (163–176)	170 (164–176)	171 (164–178)	167 (162–175)	*
Weight, kg	72 (63–81)	75 (65–84)	76 (67–85)	71.5 (63–83)	*
BMI, kg/m^2^	24.8 (22.7–27.4)	25.5 (23.3–28.3)	25.5 (23.4–28.4)	25.9 (23.1–28.9)	*

*NT-proBNP: NS (p = 0.488) when comparing NBP vs HTN grade 1.Significant when comparing HTN grade1 vs 2 p = 0.002, Significant when comparing HTN grade 2 vs 3 HTN, p = 0.01.

*CHA2DS2-VASc, NS when comparing HTN grade 1 vs 2, p = 0.681 and NS when comparing BP vs HTN grade 1, p = 0.681 and significant when comparing HTN grade 2 vs 3, p = 0.023.

*Systolic BP, Significant when comparing all the groups, p < 0.001.

*Diastolic BP, Significant when comparing all the groups, p < 0.001.

*Height, Significant when comparing NBP vs HTN1, p = 0.019. Significant when comparing HTN 1 vs HTN 2, p = 0.035, Significant when comparing HTN2 vs HTN 3, p < 0.001.

*Weight, Significant when comparing NBP vs HTN 1, p < 0.001, HTN 1 vs HTN 2, p = 0.019, and HTN 2 vs HTN 3, p < 0.001.

*BMI, Significant when comparing NBP vs HTN 1, p < 0.001, NS when comparing HTN 1 vs HTN 2, p = 0.739, and HTN 2 vs HTN 3, p = 0.959.

Vascular disease: MI and peripheral artery disease. NS = non-significant. AF, atrial fibrillation; HTN, hypertension; NT-proBNP; N-terminal pro-B-type natriuretic peptide; BP, blood pressure; NBP, Normal blood pressure; European Society of Cardiology Guidelines, ESC Guidelines; transient ischemic attack, TIA.

When comparing NT-proBNP in the different hypertension-grades with screening-detected AF, there was a trend towards higher NT-proBNP levels, but there was no significant difference between the groups (Fig. 1B and Table 3)*.*

### 3.4 Screening-detected AF in elevated systolic blood pressure

Screening-detected AF was most common in normotensive participants with increased NT-proBNP (n = 90/1922, 4.7 %), followed by those with high SBP (≥140 mm Hg) and an increased NT-proBNP (≥125 ng/L), (n = 65/1741, 3.7 %). In participants with normal blood pressure and low NT-proBNP, only two developed screening-detected AF (n = 2/1444, 0.1 %). Finally, in the group with increased blood pressure, but normal NT-proBNP, no participants developed AF. There was a significant difference between all the groups, p < 0.001.

There was no significant difference regarding screening-detected AF among participants with increased SBP (n AF = 82/3169, 2.6 %) compared to normotensive participants (n AF = 72/3044, 2.3 %), p = 0.573. There was no gradual increase of newly detected AF in hypertension-grades, p = 0.376 (Table 3)*.*

## 4 Discussion

In this sub-study of the STROKESTOP II-trial we set out to investigate the levels of NT-proBNP in elderly individuals in relation to hypertensive disease. In addition, we aimed to determine if the incidence of screening-detected AF increases with high blood pressure and NT-proBNP levels, compared to normal blood pressure/NT-proBNP levels. We show that NT-proBNP is associated with hypertensive disease and with levels of measured SBP. Furthermore, we show that elevated NT-proBNP levels is a strong predictor of AF regardless of high blood pressure, and that the risk for screening-detected AF is very low in participants with normal blood pressure and low NT-proBNP.

### 4.1 NT-proBNP in hypertensive disease

NT-proBNP was significantly higher in those with hypertension compared to non-hypertension. High blood pressure/hypertension is believed to increase myocardial stretch, which might increase the release of natriuretic peptides in the ventricles and atria [10]. A potential confounder of these results is the higher body weights seen in the hypertension group compared to the non-hypertensives, which is normally correlated with lower NT-proBNP levels [27], and could potentially decrease a difference in NT-proBNP between the groups. Despite this, NT-proBNP remained elevated in the hypertension-group compared to the non-hypertension group, in the present study.

Another explanation for higher NT-proBNP in the hypertension group, could potentially be the burden of cardiovascular comorbidities in those with hypertension diagnosis compared to non-hypertension. When adjusted for comorbidity, including removing patients with known heart failure from our study group, the difference in NT-proBNP still remained between the hypertension and non-hypertension group. In addition, hypertension itself increases the risk for increased filling pressure and heart failure, which might have remained undiagnosed, and could be seen as an increased level of NT-proBNP.

When analyzing hypertension and SBP as contributors to higher NT-proBNP levels, higher blood pressure had a larger impact on NT-proBNP than a diagnosis of hypertension in the regression models (Supplementary Table B.2). This means that NT-proBNP levels could potentially vary depending on blood pressure levels and affect the results, since higher blood pressure could be associated with elevated NT-proBNP levels. NT-proBNP is believed to promote vessel wall stress due to volume expansion in higher blood pressure, as an effect from disturbed sodium hemostasis and norepinephrine release, among many other factors [27], [28].

### 4.2 NT-proBNP in atrial fibrillation

No significant difference in NT-proBNP was observed in the screening-detected AF group, regardless of an established diagnosis of hypertension or not, nor with regards to hypertensive levels. This could be due to the small sample size with screening-detected AF (n = 154). In addition, individuals with AF have an increased NT-proBNP level at baseline and there could be a threshold effect, which might mitigate the effect on NT-proBNP.

### 4.3 NT-proBNP in hypertension grades

In the hypertension-grade group without screening-detected AF, NT-proBNP increased gradually with the grade of hypertension once blood pressure was above > 140 mmHg. This was still the case when adjusting for confounding factors. Elevated NT-proBNP in this group could also be explained by a threshold effect.

### 4.4 NT-proBNP and blood pressure as predictors of AF

In this study, both elevated NT-proBNP levels and higher blood pressure was associated with higher risk of incident AF, but the strongest predictor of AF was NT-proBNP alone. Our results suggest that elevated NT-proBNP levels are a more direct indicator of atrial strain or underlying cardiac dysfunction, which are closely linked to AF development. While hypertension is an important cardiovascular risk factor, NT-proBNP seems to reflect more specific pathophysiological changes, such as increased atrial pressure and myocardial stretch that can predispose to AF [10], [29], [30]. This might explain why NT-proBNP alone, without concomitant hypertension, emerged as the strongest marker. Another explanation for why elevated NT-proBNP alone seem to increase the risk of screening detected AF, could be due to the unknown comorbidities such as undiagnosed heart failure and chronic kidney disease that could have worked as confounders and contributed to AF and elevated NT-proBNP. Our results suggest that NT-proBNP can serve as a surrogate marker for identifying high-risk individuals, and that NT-proBNP reflects subclinical changes that are not easily captured by blood pressure measurements alone.

Since NT-proBNP seems to be elevated in both AF and high blood pressure, perhaps it could work as a surrogate marker and a predictor of AF, distinguishing whom to screen, in a hypertensive population. Similar results have recently been shown in Xing and Diederichsen et al, who conducted a secondary analysis of the LOOP study, analysing NT-proBNP as a predictor of AF and stroke [31]. They found that higher levels of NT-proBNP (>125 ng/L) were associated with an increased risk of AF in both the ILR-group and the control group which is similar to our findings. Increased levels of NT-proBNP were also associated with an increased risk of stroke, cardiovascular events, and death and several studies have shown similar results [3], [5], [32]. Another recent study by Schmalstieg et al [33], have similarly showed elevated NT-proBNP levels in elderly patients with AF compared to no AF. They concluded that NT-proBNP could be used to detect early AF in risk individuals but that larger trials are needed. Even if our study included a large amount of participants compared to Schmalstieg et al, the AF detection was similarly low (2,5%), and findings concerning the AF group was based on a small sample size, which makes it difficult to draw any firm conclusions. In another sub-analysis of the LOOP-study, higher SBP (≥150 mmHg) was associated with an increased risk of longer AF episodes (≥24 h) detected by ILR compared to lower blood pressure [34]. We also did a sub analysis of patients with a SBP > 150 mmHg, and NT-proBNP > 125 ng/l, compared to those with NT-proBNP < 125 ng/L + BP < 150 mmHg, and saw that although AF detection was numerically higher (n = 35/1042, 3.3 % vs n = 122/5188, 2.3 %) we could not detect a significant difference between groups, p = 0.058.

Our sub-study did not confirm a positive correlation between higher blood pressure and increased AF risk, and the overall AF-detection was low (2,5%). Several studies, on the contrary, have shown a positive correlation between higher SBP and elevated AF risk [35], [36]. The Atherosclerosis Risk in Communities-study showed that higher BP ≥ 140 mmHg was correlated with increased AF, and a risk-reduction in AF with lower blood pressure [36]. A sub-analysis of the Framingham heart study also found a positive correlation between elevated SBP and increased risk of AF, where hypertensive individuals had a potential doubled risk of developing AF compared to normotensive individuals [35]. On the contrary, Healey et al [37] reported a lower risk of AF ≥ 5 min in increasing SBP.

Furthermore, it is not certain if the risk of incident AF decreases with blood pressure-control. More studies are needed regarding NT-proBNP levels and dynamics in atrial fibrillation and hypertension, together with other cardiac/extra-cardiac conditions to understand which cutoffs/levels of NT-proBNP, that may be relevant in predicting AF. Since NT-proBNP is an easy and already established method in clinical practice, it could potentially be used as a promising predictor of AF in future screening programs.

The present study, together with previous research, motivate more extensive screening of AF in hypertensive individuals in the future, where NT-proBNP might work as a diagnostic/prognostic tool to differentiate whom to screen.

### 4.5 Limitations

The present study had several limitations, primarily the small group of screening-detected AF in every hypertension-grade, as we were likely underpowered for firm conclusions. In addition, heart rate in newly detected AF was not considered when interpreting NT-proBNP levels, which could have affected the results [38]. NT-proBNP levels < 60 ng/l and ≥ 9000 ng/L were not specified, which also could have influenced the NT-proBNP levels. Comorbidities were self-reported, which could have caused misclassification bias. Other possible confounders could be renal failure, which was not reported and considered and could also have affected the NT-proBNP levels [39].

Blood pressure was measured during office hours, and different time points of measuring blood pressure could have influenced the results. Hypertensive treatment was not considered when interpretating the results and could also have affected NT-proBNP levels. In patients with low NT-proBNP, prolonged rhythm monitoring was not performed, which could have led to detection bias. With regards to the index ECG recordings, they all were reviewed by a research nurse at the time of registration and had an automatic annotation with a high sensitivity [40]. Therefore, false-negative ECGs using both manual and algorithm screening would likely be rare. The low incidence of AF and the large overlap of NT-proBNP between groups can make the evaluation and suitability in screening for AF difficult to judge, and therefore more and larger trials are needed for assessment.

However, the large size of the present study cohort and the high number of unselected and relevant screening-population with hypertension/high blood pressure, are strengths in this sub-study. Other strengths of this study were the exclusion of participants with known AF and heart failure, since that could have interfered with the NT-proBNP levels. When adjusted for cardiovascular comorbidities, NT-proBNP still remained elevated in the hypertension group compared to the hypertension group and in the different hypertension-grades.

## 5 Conclusion

NT-proBNP levels were elevated in elderly individuals with hypertension and there is an association between NT-proBNP and increasing hypertension grades. The incidence of AF was highest in normotensive participants with elevated NT-proBNP (>125 ng/L), followed by participants with higher SBP (≥140 mmHg) and elevated NT-proBNP (>125 ng/L), compared to individuals with normal SBP/NT-proBNP levels. NT-proBNP might in the future be used as an additive risk marker in patients with hypertension to screen for AF, but more and larger studies are needed.

**Funding**

This study is funded by an unrestricted research grant by Roche Diagnostics Ltd. The funders have had the opportunity to read the paper prior to publication.

Dr Svennberg is supported by the Stockholm County Council (Clinical researcher appointment), the Swedish Research Council (DNR 2022–01466), the Swedish Heart and Lung foundation and CIMED.

## CRediT authorship contribution statement

**Gina Sado:** Writing – review & editing, Writing – original draft, Visualization, Software, Project administration, Methodology, Investigation, Formal analysis, Data curation, Conceptualization. **Katrin Kemp Gudmunsdottir:** Writing – review & editing, Supervision, Methodology, Formal analysis, Data curation, Conceptualization. **Carl Bonander:** Writing – review & editing, Supervision, Formal analysis, Conceptualization. **Mattias Ekström:** Writing – review & editing, Supervision, Conceptualization. **Johan Engdahl:** Writing – review & editing, Supervision, Resources, Funding acquisition, Formal analysis, Conceptualization. **Emma Svennberg:** Writing – review & editing, Visualization, Validation, Supervision, Resources, Project administration, Funding acquisition, Formal analysis.

## Declaration of competing interest

The authors declare the following financial interests/personal relationships which may be considered as potential competing interests: GS reports no disclosures. KKG has received research grants from Roche Diagnostics and the Swedish Heart Lung Foundation and lecture fees from Roche Diagnostics and Boehringer Ingelheim. ME reports lecture/consulting fees from Amarin, Amgen, Sanofi AB and research grants from Novartis foundation from medical-biological research. JE has received consultant or lecture fees from Roche Diagnostics, Pfizer, Bristol Myers Squibb, Boehringer Ingelheim, Piotrode and Philips, and research grants from the Swedish Research Council, The Swedish Heart Lung Foundation, The Swedish Innovation Agency, and The Stockholm Region. ES reports lecture/consulting fees (institutional) from Abbott, Astra Zeneca, Bristol-Myers Squibb-Pfizer and Johnson & Johnson. CB and GS reports no COI.
