# Supplementary material for: The role of NT-proBNP in screening for atrial fibrillation in hypertensive disease
Source: Int J Cardiol Heart Vasc. 2024 Nov 8;55:101549. doi: 10.1016/j.ijcha.2024.101549 (PMC11795693; doi:10.1016/j.ijcha.2024.101549)
Supplement: Supplementary Data 1 [file mmc1.docx]

**Supplementary material**

**Supplementary Figure A.1. Study flow chart.** Number of participants with valid NT-proBNP levels after exclusion of patients with heart failure, known atrial fibrillation and the exclusion of participants due to death/emigration/non-repliers. AF, atrial fibrillation; NT-proBNP, N-terminal pro-B-type natriuretic peptide; CHF, congestive heart failure.

|  |  |  |  |  |  |
| --- | --- | --- | --- | --- | --- |
| **Supplementary table A.1. Characteristics and median NT-proBNP in 75/76 year-old participants with different hypertension grades without screening-detecting AF** | | | | | |
|  | **NBP  n=3281** | **HTN grade 1  n=1944** | **HTN grade 2  n=712** | **HTN grade 3  n=143** | **p(NBP vs HTN grades)** |
| **Sex, n (%)** |  |  |  |  |  |
| Female gender | 1934 (59%) | 1020 (52.5%) | 347 (48.7%) | 91 (63.6%) | p<0.001 |
|  |  |  |  |  |  |
| **Comorbidities, n (%)** |  |  |  |  |  |
| Diabetes mellitus, n (%) | 360 (11%) | 215 (11.1%) | 65 (9,1%) | 15 (10.5%) | p=0.497 |
| Prior stroke/TIA, n (%) | 256 (7.8%) | 135 (6.9%) | 43 (6%) | 5 (3.4%) | p=0.097 |
| Vascular disease, n (%) | 197 (6%) | 108 (5.6%) | 38 (5.3%) | 8 (5.6%) | p=0.854 |
| Hypertension, n (%) | 1395 (41.3%) | 1149 (59.3%) | 479 (67.5%) | 108 (76.1%) | p<0.001 |
|  |  |  |  |  |  |
| **Median, (IQR)** |  |  |  |  |  |
| NT-proBNP, ng/L | 142(81-244) | 146(85-251) | 165 (88-283) | 213 (117-363) | * |
| CHA_2_DS_2_-VASc score | 3 (3-4) | 3 (3-4) | 3 (3-4) | 4 ( 3-4) | ** |
| Systolic BP mmHg | 128 (120-134) | 148 (143-153) | 166 (162-171) | 185 (182-191) | p<0.001 |
| Diastolic BP, mmHg | 77(72-81) | 85(79-91) | 92(84-97) | 97(90-103) | p<0.001 |
| Height (cm) | 169 (163-176) | 170 (163-176) | 171 (164-178) | 167 (161-175) | ** |
| Weight (kg) | 72 (63-81) | 75 (65-85) | 76 (66-85) | 71 (63-83) | ** |
| BMI (kg/m^2^) | 24.8(22.7-27.4) | 25.5(23.3-28.3) | 25.4(23.4-28.4) | 25.9 (23.1-28.8) | ** |
|  |  |  |  |  |  |
| ***NT-proBNP:** NS (p=0.121) when comparing NBP vs HTN grade 1.Significant when comparing HTN grade1 vs 2 p=0.012, and HTN grade 2 vs 3 HTN, p = 0.003 | | | | | |
| ****CHA2DS2-VASc,** NS when comparing HTN grade 1 vs 2, p=0.717.Significant when comparing NBP vs HTN grade 1, p<0,001 and HTN grade 2 vs 3, p=0.008. | | | | | |
| **Systolic BP**, Significant when comparing all the groups | | |  |  |  |
| **Diastolic BP**, Significant when comparing all the groups | | |  |  |  |
| ****Height**, Significant when comparing NBP vs HTN1, p=0.018. NS when comparing HTN 1 vs HTN 2, p= 0.058, Significant when comparing HTN2 vs HTN 3, p<0.001. | | | | | |
| ****Weight,** Significant when comparing NBP vs HTN 1, p<0.001. NS when comparing HTN 1 vs HTN 2, p=0.130, Significant when comparing HTN 2 vs HTN 3, p=0.012 | | | | | |
| ****BMI,** Significant when comparing NBP vs HTN 1, p<0.001, NS when comparing HTN 1 vs HTN2, p=0.735, NS when comparing HTN 2 vs HTN 3, p=0.971 | | | | | |
| NS = non-significant. AF, atrial fibrillation; HTN, hypertension; NT-proBNP; N-terminal pro-B-type natriuretic peptide; BP, blood pressure; NBP, Normal blood pressure; European Society of Cardiology Guidelines, ESC Guidelines; transient ischemic attack, TIA | | | | | |

| **Supplementary table B.2. Multiple linear regression results for the association between logNT-proBNP (outcome) and hypertension (binary classification and in grades).** | | | |
| --- | --- | --- | --- |
|  | Coefficients (std. errors) | | |
| Characteristic | Model 1 | Model 2 | Model 3 |
| Hypertension (yes/no) | .075***  (.023) | .036  (.023) | - |
| Hypertension grades |  |  |  |
| Normal blood pressure | - | - | 0 (reference) |
| HTN 1 | - | - | .072**  (.024) |
| HTN 2 | - | - | .166***  (.034) |
| HTN 3 | - | - | .329***  (.072) |
|  |  |  |  |
| Adjustment variables |  |  |  |
| Sex (Male) | -.273***  (.032) | -.292***  (.032) | -.275***  (.032) |
| Weight (kg) | -.010***  (.001) | -.010***  (.001) | -.010***  (.001) |
| Height (cm) | .011***  (.003) | .012***  (.002) | .011***  (.003) |
| Weight*Sex | .004  (.002) | .004  (.002) | .004  (.002) |
| Height*Sex | .002  (.004) | .002  (.004) | .002  (.004) |
| Previous stroke/TIA | .049  (.047) | .056  (.042) | .071  (.042) |
| Screening-detected AF | 1.022***  (.069) | 1.041***  (.068) | 1.032***  (.068) |
| Diabetes | -.016  (.036) | -.038  (.036) | .005  (.035) |
| Vascular disease | .491***  (.047) | .480***  (.047) | .506***  (.0466) |
| Systolic blood pressure | - | .007***  (.001) | - |
| Diastolic blood pressure | - | -.01***  (.001) | - |
| *Notes:* *p≤0.05, **p≤0.01, ***p≤0.001. Standard errors in parentheses. Hypertension; HTN. Vascular disease: Myocardial infarction and peripheral vascular disease. Weight and height were centered at their sample means to facilitate interpretation of the coefficients involved in the interaction terms. | | | |
